# Launching an Interactive Cancer Projects Map: A Collaborative Approach to Global Cancer Research and Program Development

**DOI:** 10.1200/JGO.2015.000034

**Published:** 2015-09-23

**Authors:** Edward L. Trimble, Ali A. Chisti, Jane A. Craycroft, Kalina Duncan, Manaswi Gupta, Daniel Gutierrez, Ilyana Rosenberg, Nour Sharara, Sudha Sivaram, Hillary M. Topazian, Jing Jing Wang, Makeda J. Williams, Franklin W. Huang, Ami S. Bhatt

**Affiliations:** **Edward L. Trimble**, National Cancer Institute, Center for Global Health, Bethesda, MD; **Ali A. Chisti**, Global Oncology, Boston, MA, and University of Hawaii, Honolulu, HI; **Jane A. Craycroft**, Global Oncology, Boston, MA, and Dana-Farber Cancer Institute, Boston, MA; **Kalina Duncan**, National Cancer Institute, Center for Global Health, Bethesda, MD; **Manaswi Gupta**, Global Oncology, Boston, MA, and Broad Institute of MIT and Harvard, Cambridge, MA; **Daniel Gutierrez**, Global Oncology, Boston, MA; **Ilyana Rosenberg**, Global Oncology, Boston, MA; **Nour Sharara**, Global Oncology, Boston, MA; **Sudha Sivaram**, National Cancer Institute, Center for Global Health, Bethesda, MD; **Hillary M. Topazian**, National Cancer Institute, Center for Global Heatlh, Bethesda, MD; **Jing Jing Wang**, Global Oncology, Boston, MA; **Makeda J. Williams**, National Cancer Institute, Center for Global Health, Bethesda, MD; **Franklin W. Huang**, Global Oncology, Boston, MA, and Dana-Farber Cancer Institute, Boston, MA; **Ami S. Bhatt**, Global Oncology, Boston, MA, and Stanford University, Stanford, CA.

Despite living in a connected world, many research projects are developed with a so-called “convenience bias,” resulting in partnerships based on existing professional networks and collaborations. In addition, community-based cancer control programs are often implemented independently to address specific urgent and existing needs. Consequently, these projects and programs do not benefit as much as they could from best practices and protocols developed through other long-term engagements. As the global burden of cancer increases, cancer researchers and program managers are likely to benefit from a more complete understanding of ongoing work in cancer control. The limited availability of tools for collaboration and sharing of best practices have resulted in a call to action for the scientific community to partner on the development of a platform that provides knowledge of existing resources and expertise.

**Figure 1:**
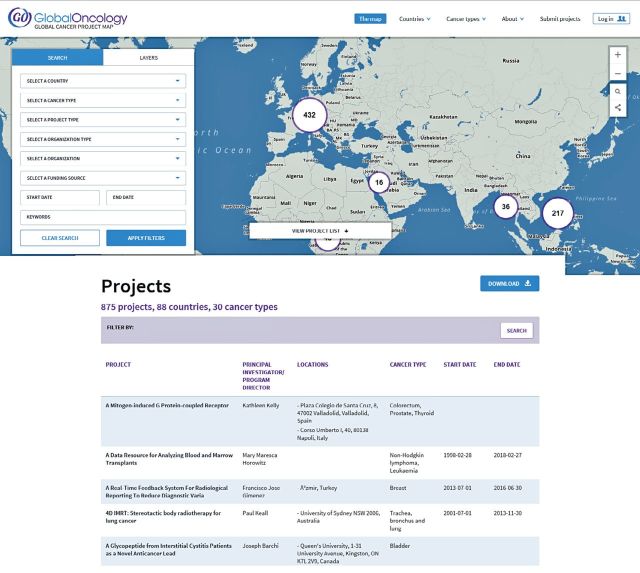
Homepage for the Global Cancer Project Map.

Global Oncology (GO), a nonprofit organization, and the Center for Global Health (CGH) at the National Cancer Institute (NCI) have developed a Web-based tool that facilitates planning of research, training opportunities, and community-based programs in cancer control. This tool, called the Global Cancer Project Map (GCPM; http://gcpm.globalonc.org), is an interactive Web site that enables viewers to locate cancer projects and research programs as displayed on a world map (Figure 1). GCPM currently displays more than 800 projects of more than 620 investigators working at more than 160 institutions in 88 countries (Table 1) . The inaugural version of the GCPM was officially launched on March 25, 2015, at the Symposium on Global Cancer Research, sponsored by NCI, the Consortium of Universities for Global Health (CUGH), and the Dana-Farber Cancer Institute.

**Table 1:** Current GCPM Portfolio Breakdown and Descriptive Fields

Funding Source	Search Filters	Statistics Layers*	Project	
			Type	No.
NCI FY2013†	Country	Incidence‡	Basic research	280
ASCO FY2014	ICD cancer type (31 cancer categories)	Prevalence‡	Clinical research	86
ASCO Conquer Cancer Foundation	Project type	Mortality‡	Population-based research	79
Dana-Farber/Harvard Cancer Institute	Organization type	Disability-adjusted life-year§	Training	63
Fred Hutchinson Cancer Research Center/University of Washington Cancer Consortium	Organization	Human development index‖	Capacity building	25
Moffitt Cancer Center	Funding source		Cancer prevention	17
Sidney Kimmel Comprehensive Cancer Center	Start date		Cancer screening	10
The University of Texas MD Anderson Cancer Center	End date Keywords (full text search of entire project entry)		Cancer detectione	59
			Cancer treatment	21
			Cancer surveillance	17
			cancer registries Palliative care	5

Abbreviations: ASCO, American Society of Clinical Oncology; GCPM, Global Cancer Project Map; ICD, International Classification of Diseases; NCI, National Cancer Institute.

* All cancer-related statistics are for all cancer types using age-standardized rates.

† FY2014 data to be uploaded in September will include approximately 1,280 projects.

‡ GLOBOCAN 2012, International Agency for Research on Cancer.

§ Global Burden of Disease, Institute for Health Metrics and Evaluation, 2010.

‖ Human Development Reports, United Nations Development Programme.

The data initially used to populate the GCPM consisted of projects supported by NCI extramural international awards, including direct grants to foreign institutions. Data collection and upload have also begun on the international endeavors of NCI-designated cancer centers and other international partners, such as the American Society of Clinical Oncology and the Union for International Cancer Control. This resource establishes a broad base of global cancer-related projects that will continually expand as data are added to the site.

At present, users can access the Web site from anywhere in the world at no cost. Designed to be streamlined and user-friendly, the site is optimized for database performance, search speed, and the ability to visualize projects and potential collaborators. Projects can be searched by keyword, cancer type, project type (eg, basic research, training, cancer screening), and country. Additional descriptive statistics such as the Human Development Index or cancer incidence and prevalence figures can easily be overlaid on the map to identify disparities between relative research investment and cancer epidemiology. GCPM offers the potential to be used as a resource for current cancer-related project information, uniting and educating stakeholders at all levels in their cancer control efforts.

It is projected that the linkages facilitated by GCPM will catalyze a process of addressing cancer control in parts of the world where we see overwhelming inequities and disparities while minimizing duplication of efforts, which will lead to more efficient use of resources. Global oncology literature comprises only a small portion of published work and often faces a lengthy process to enter the public domain. GCPM makes information about these ongoing projects freely available to allow cancer researchers and program managers access to a platform where information on global cancer work can be exchanged and ideas can be developed. This economy of scale has great utility in hastening progress in this rapidly developing field. Ongoing and planned enhancement of Web site content with additional projects from partnering governmental, nongovernmental, and academic sources will help facilitate partnerships and collaborations in areas that can improve research and control and contribute to the overall reduction of the global burden of cancer.

